# Supplementary material for: Jarid2 binds mono-ubiquitylated H2A lysine 119 to mediate crosstalk between Polycomb complexes PRC1 and PRC2
Source: Nat Commun. 2016 Nov 28;7:13661. doi: 10.1038/ncomms13661 (PMC5133711; doi:10.1038/ncomms13661)
Supplement: Supplementary Information — Supplementary Figures 1 - 9 and Supplementary Table 1 [file ncomms13661-s1.pdf]

Supplementary Figure 1

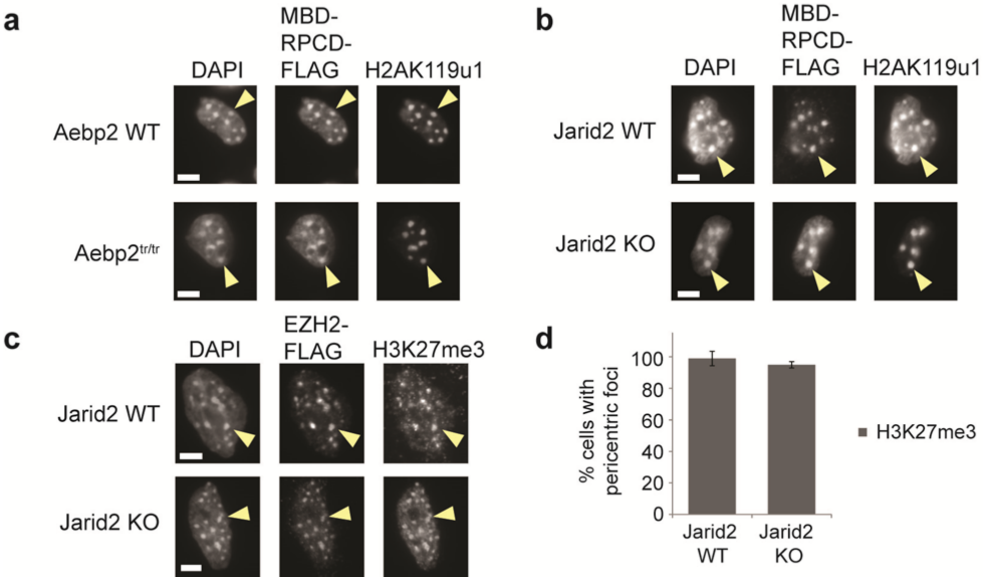

Supplementary Figure 1: H2AK119u1 and H3K27me3 domains at pericentric heterochromatin in *Aebp2*$^{tr/tr}$, *Jarid2* KO and complemented *Jarid2* KO mESCs.

H2AK119u1 in *Aebp2*^{WT} and *Aebp2*$^{tr/tr}$ (**A**) and *Jarid2* WT and KO (**B**) is observed at the pericentric heterochromatin (PCH) upon transient expression of MBD-RPCD. Arrowhead indicates a PCH focus. Scale bar is 5 µm. (**C**) Transient expression of MBD-Ezh2 in *Jarid2* WT and KO mESCs and immunofluorescence staining for H3K27me3. In both mESCs lines H3K27me3 is visualised at PCH (arrowhead). Scale bar is 5 µm. (**D**) Quantification of cells in figure (**C**). Error bars display standard deviation. A minimum of 300 cells were counted in 3 biological repeats.

Supplementary Figure 2

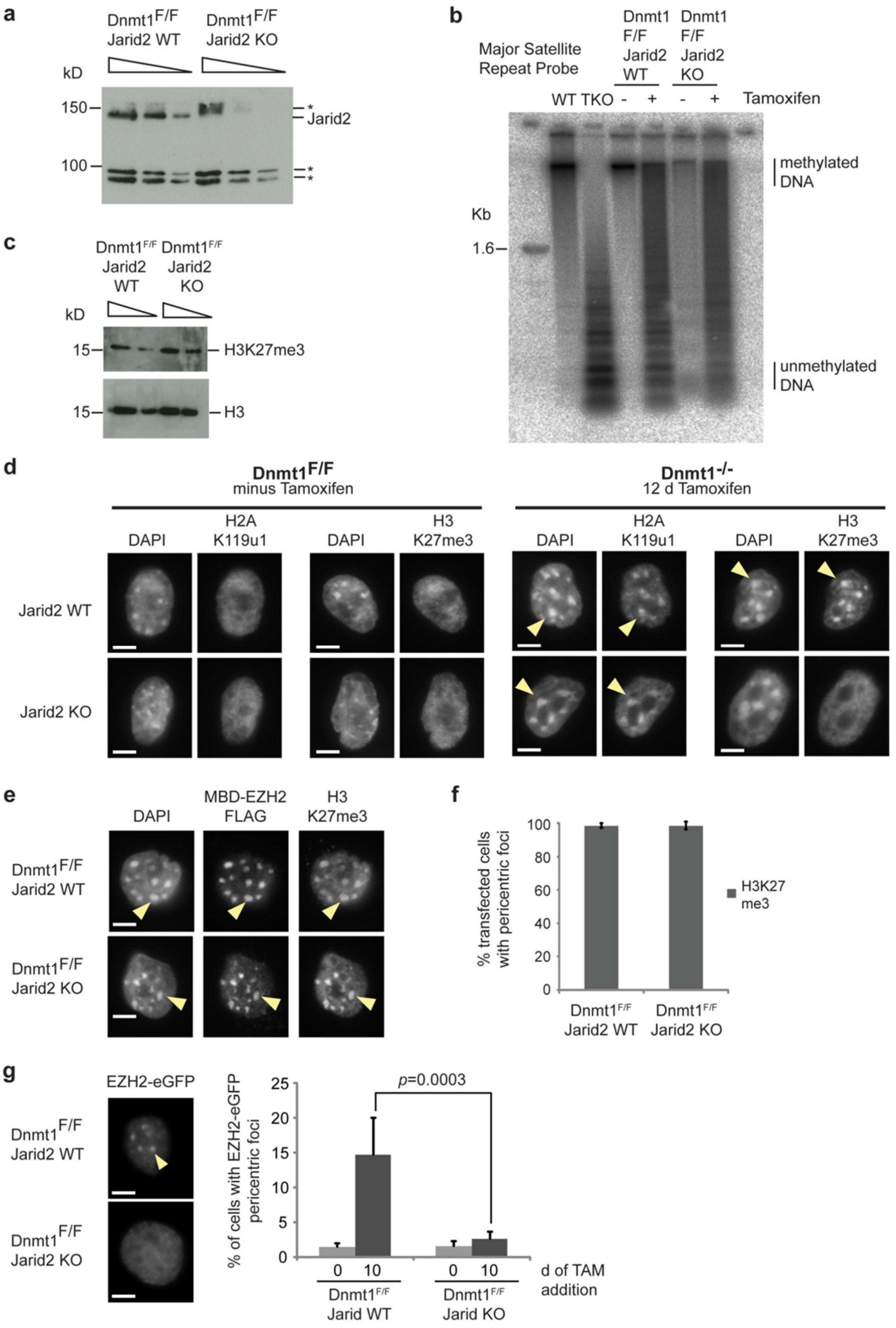

31

Supplementary Figure 2: Characterisation of *Jarid2* KO mESCS in *Dnmt1*$^{F/F}$ background. (**A**) Immunoblot of whole cell extracts from mESCs showing depletion of JARID2 in the *Jarid2* KO line. Asterisks indicate species cross-reacting with the antibody. (**B**) Southern blot showing loss of DNA methylation at major satellite repeats upon tamoxifen induction of Dnmt1$^{F/F}$ mESCs. Genomic DNA from control mESCs :WT and TKO (*Dnmt1*$^{-/-}$, *Dnmt3a*$^{-/-}$ *Dnmt3b*$^{-/-}$) or test mESCs : *Dnmt1*$^{F/F}$ *Jarid2* WT or KO, plus or minus tamoxifen (to induce deletion of Dnmt1) was digested with the methylation-sensitive HpyCH41V and the blot was probed for major satellite repeats. (**C**) Immunoblot of histone extracts of *Dnmt1*$^{F/F}$ *Jarid2* WT or KO probed for H3 and H3K27me3. (**D**) Immunofluorescence staining of *Dnmt1*$^{F/F}$ *Jarid2* WT and *Dnmt*$^{F/F}$ *Jarid2* KO mESCs for H2AK119u1 and H3K27me3 either without tamoxifen induction (left) or with 12 days tamoxifen induction (right). Arrowhead indicates a pericentric heterochromatin focus. (**E, F**) Transient expression of MBD-Ezh2 in *Dnmt1*$^{F/F}$ *Jarid2* WT and KO mESCs and immunofluorescence staining for H3K27me3 (**E**), quantified in graph (**F**). In both mESC lines H3K27me3 is visualised at PCH (arrowhead). (**G**) Stable EZH2-GFP mESC lines were created in both *Dnmt1*$^{F/F}$ *Jarid2* WT and *Dnmt*$^{F/F}$ *Jarid2* KO backgrounds and localisation to PCH was assessed by GFP fluorescence (left) and quantified (right). P value Student's t-test, unpaired. Scale bars represent 5 µm. Arrowheads indicate a PCH domain. In all experiments a minimum of 300 cells were counted in 3 biological repeats. Error bars indicate standard deviation.

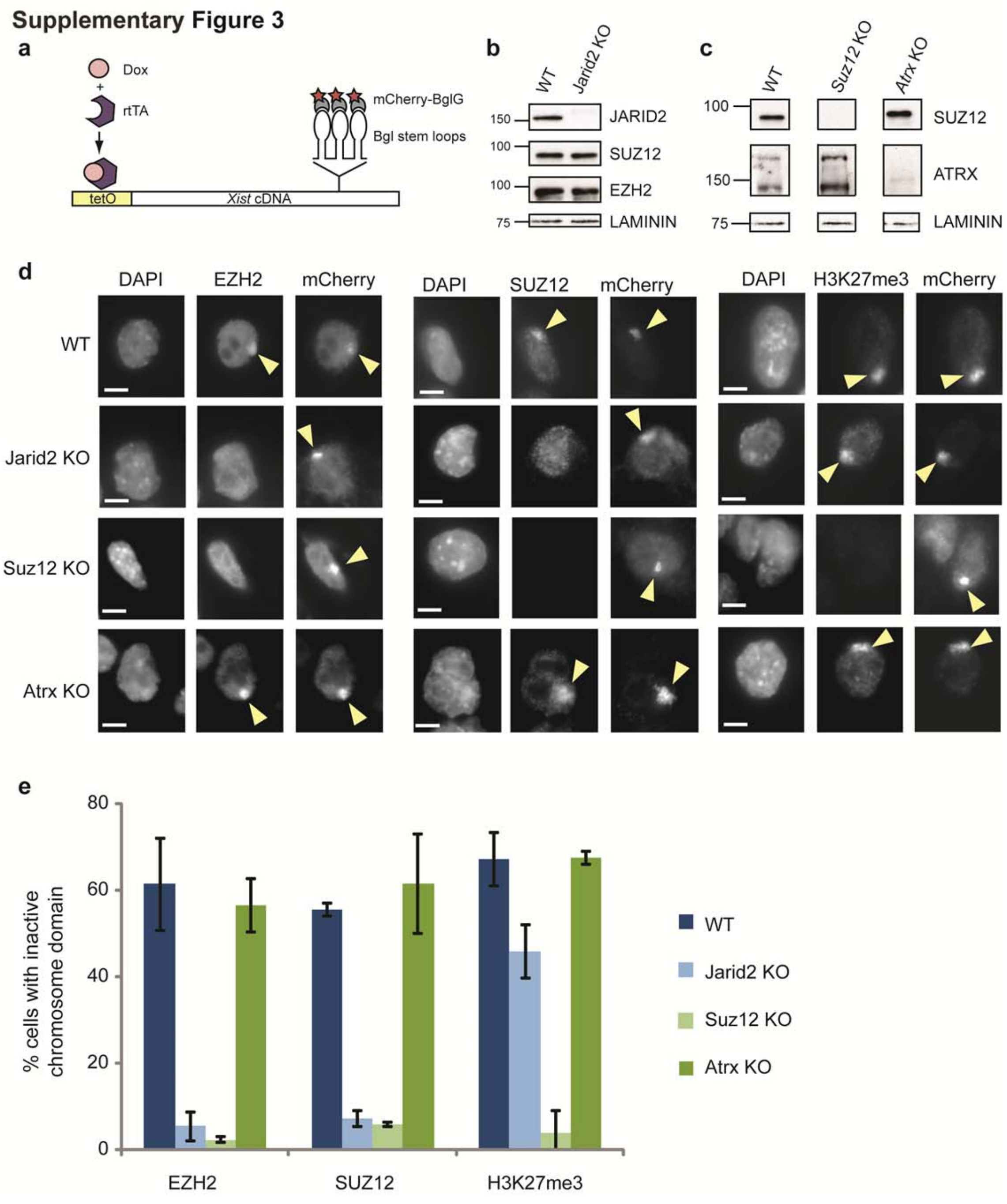

Supplementary Figure 3: Deletion of Jarid2 diminishes Xist RNA dependent recruitment of PRC2. (A) The mESC cell line BglXist1 has a doxycyclin-inducible *Xist* transgene which contains BglG stem loops on an autosome. The binding of Bgl-mCherry identifies the *Xist* coated chromosome. (**B**) Immunoblot showing levels of the indicated

proteins in the *Jarid2* KO line. (**C**) Immunoblot showing levels of the indicated proteins in *Suz12* and *Atrx* KO cell lines. (**D**) Immunofluorescence for H3K27me3 and PRC2 components EZH2 and SUZ12 in WT, *Jarid2, Suz12* and *Atrx* KO mESCs after induction of *Xist*. Arrowhead indicates an inactive chromosome focus. (**E**) Quantification of foci observed. A minimum of 300 cells were counted in 3 biological repeats. Error bars indicate standard deviation.

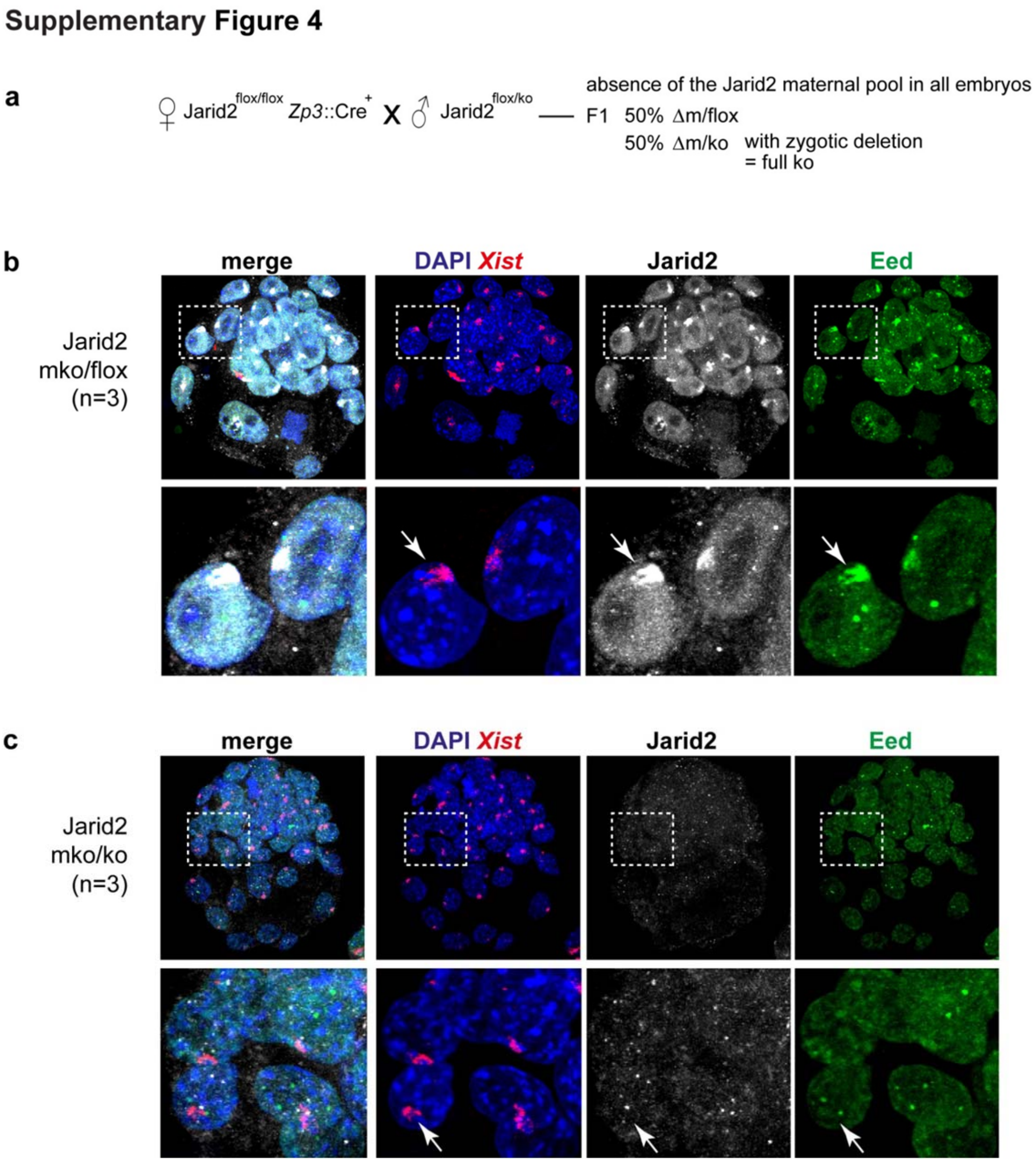

Supplementary Figure 4: JARID2 absence disrupts EED recruitment to the inactive X during preimplantation development. (**A**) Schematic of mating scheme to generate complete *Jarid2* knockout embryos. (**B**) Immunofluorescence and *Xist* RNA FISH of heterozygote maternal *Jarid2* KO/paternal *Jarid2*$^{F/F}$ (mko/flox) embryos at late morula/blastocyst stage. JARID2 (grey) and EED (green) recruitment on *Xist*-coated chromosomes (red; white arrow). (**C**) As (**B**), but homozygous *Jarid2* KO embryos

(maternal *Jarid2* KO/paternal *Jarid2*$^{-/-}$ (mko/ko). In (**B**) and (**C**), lower panel indicates zoom from the selected area in upper panel. DAPI is counterstained in blue. Scale bar is 10 µm.

Supplementary Figure 5

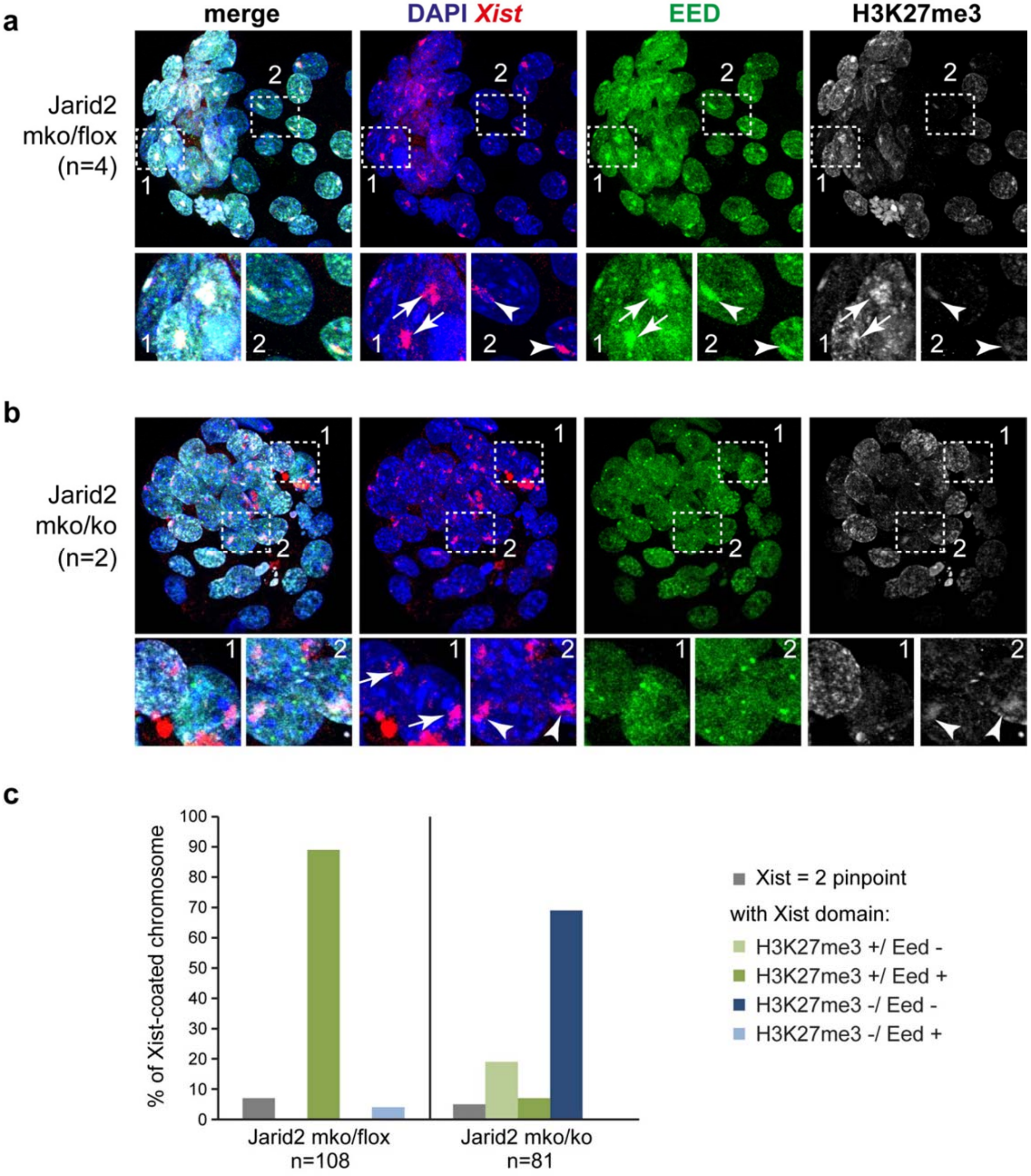

Supplementary Figure 5: Absence of JARID2 disrupts PRC2 recruitment to the inactive X during preimplantation development. (**A**) Immunofluorescence and *Xist* RNA FISH of heterozygote maternal *Jarid2* KO/paternal *Jarid2*$^{F/F}$ (mko/flox) embryos at blastocyst stage. EED (green) recruitment and H3K27me3 enrichment (grey) on *Xist*-coated

chromosomes (red; white arrow) are shown. Lower panel indicates zoom from the selected area in upper panel. DAPI is counterstained in blue. Scale bar is 10 µm. (**B**) As (**A**), but with homozygous *Jarid2* KO embryos (maternal *Jarid2* KO/paternal *Jarid2*$^{-/-}$ (mko/ko)). (**C**) % of *Xist*-coated chromosomes counted from embryos as in (**A**) or (**B**). n represents numbers of blastomere cells counted.

Supplementary Figure 6

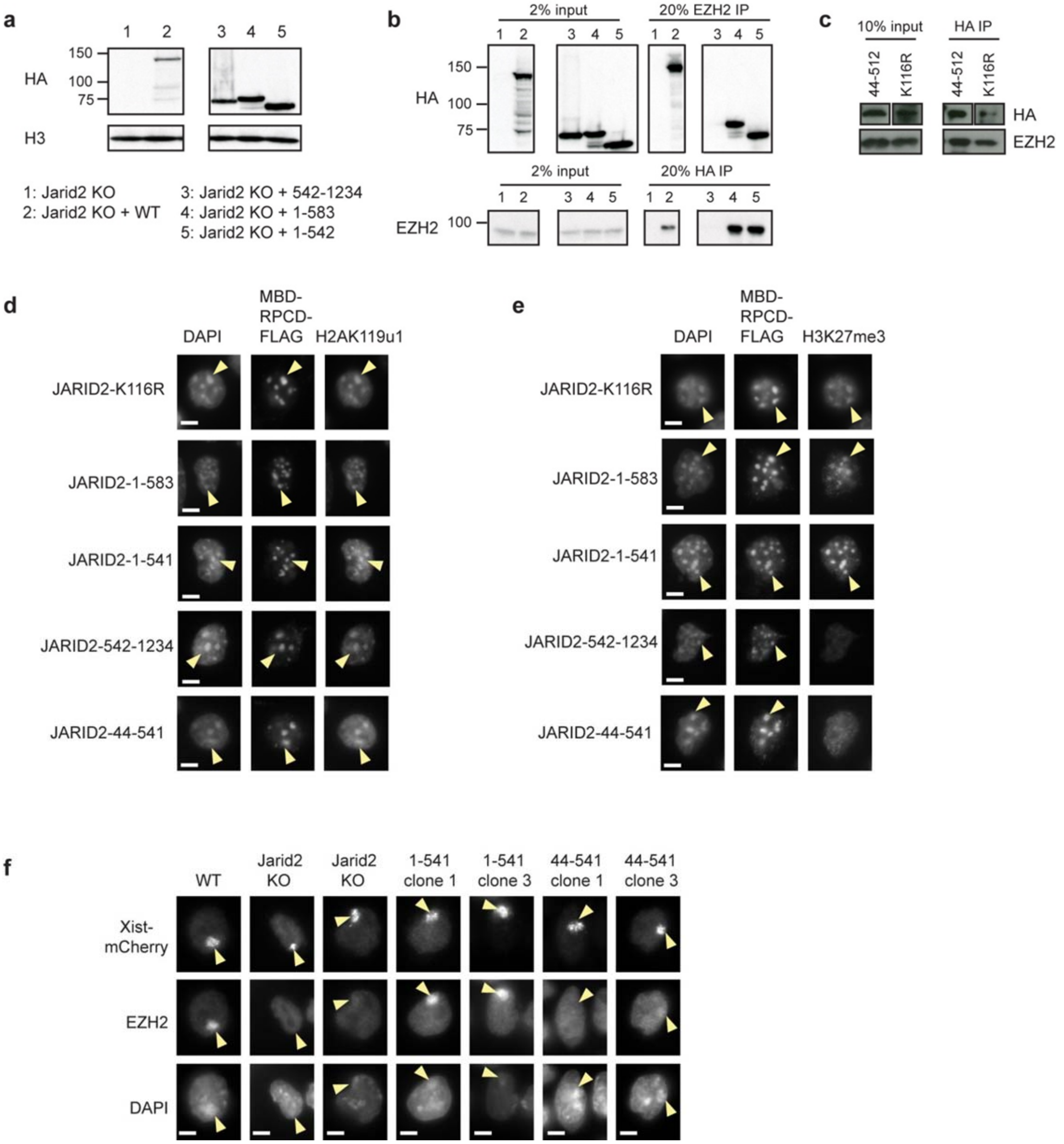

Supplementary Figure 6: The N-terminus and UIM of Jarid2 complement PRC2 recruitment to H2AK119u1 chromatin. (**A**) Immunoblot of whole cell extracts of complemented *Jarid2* KO cell lines normalised for H3. (**B**) Immunoblot of reciprocal co-immunoprecipitation of HA-tagged JARID2 fragments and EZH2. (**C**) Co-immunoprecipitation of EZH2 with HA-tagged Jarid2 44-512 and Jarid2 K116R (**D**) H2AK119u1 is observed at the PCH upon expression of MBD-RPCD in complemented

Jarid2 KO mESCs. (**E**) H3K27me3 is observed at the PCH after complementation with deletion constructs containing the N-terminal region, but not with a construct containing the C-terminus or with the construct JARID2-44-541. Quantification is shown in Fig. 3B. Scale bar is 5 µm. (**F**) Examples illustrating analysis of Xist dependent EZH2 recruitment in WT, *Jarid2* null and two independent cell lines complemented with JARID2 1-541 or JARID2 44-541. Quantification is shown in Fig. 3E. Examples for *Jarid2* null mESCs illustrate absent (left panel) or weak (right panel) EZH2 staining. Arrowheads indicate a position of Xist domain in all panels. Scale bars represent 5 µm.

Supplementary Figure 7

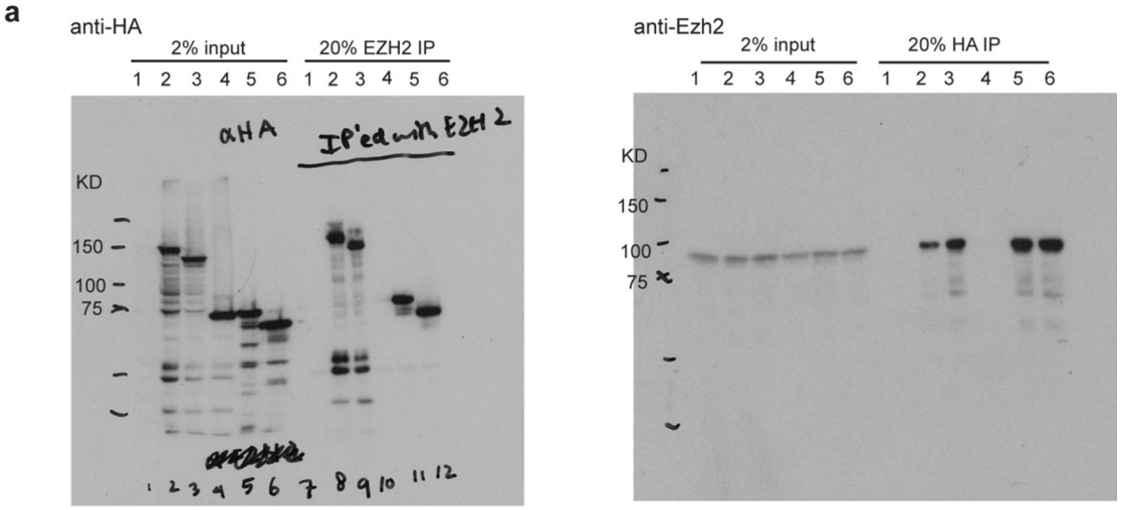

1: Jarid2 KO
2: Jarid2 KO + WT
3: Jarid2 KO + 121-1234
4: Jarid2 KO + 542-1234
5: Jarid2 KO + 1-583
6: Jarid2 KO + 1-542

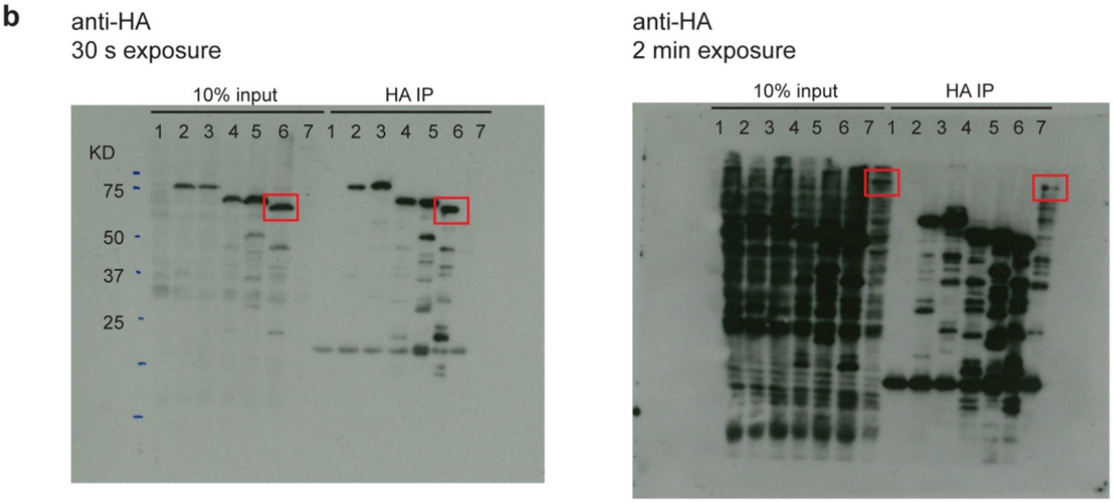

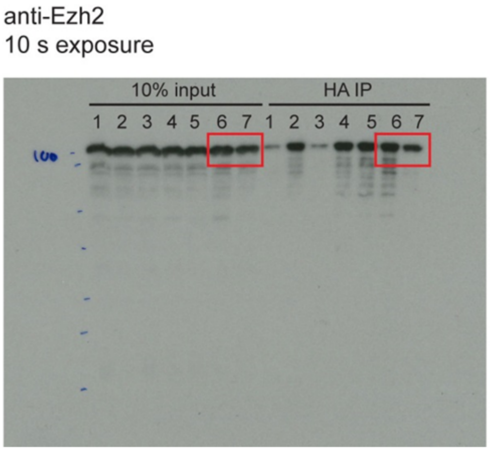

1: Jarid2 KO
2: Jarid2 KO + 1-583
3: Jarid2 KO + 542-1234
4: Jarid2 KO + 1-512
5: Jarid2 KO + 44-542
6: Jarid2 KO + 44-512
7: Jarid2 KO + FL K116R

Supplementary Figure 7: Full Immunoblots. (**A**) Complete immunoblots for the experiment shown in Supplementary Figure 6b. (**B**) Complete immunoblots for the experiment shown in Supplementary Figure 6c. The red boxes indicate the regions of the blots that have been cropped.

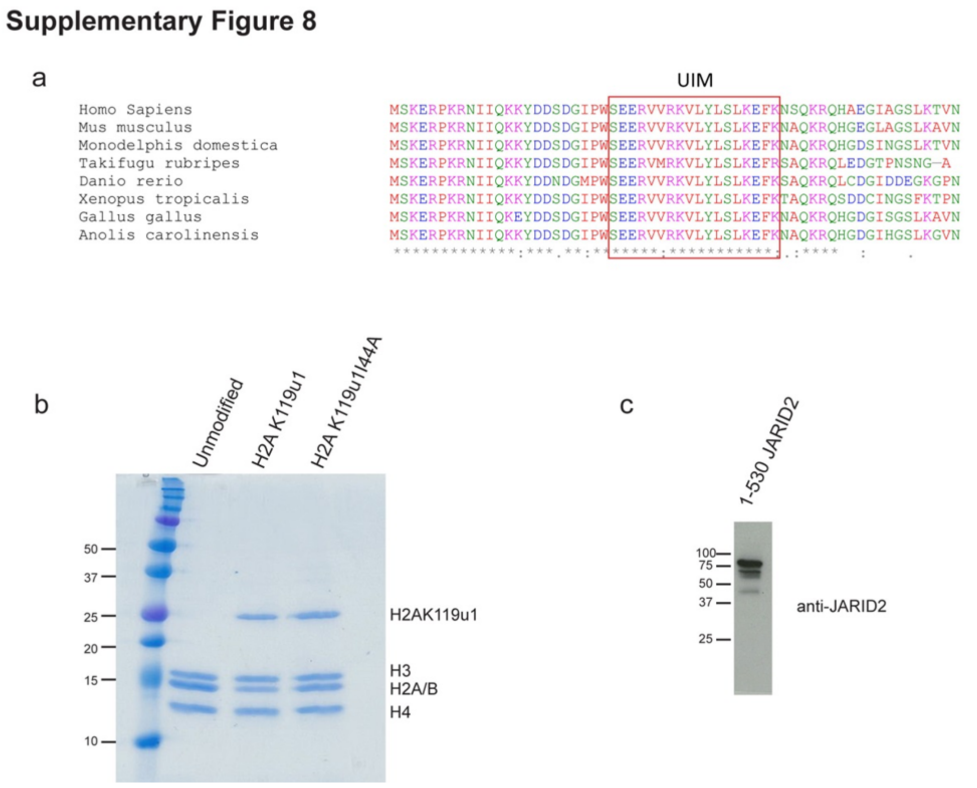

Supplementary Figure 8: Biochemical analysis of Jarid2 interaction of with H2AK119u1 nucleosomes. (**A**) Alignment of UIMs from vertebrates. (**B**) Coomassie staining of polyacrylamide gel of histone octamers reconstituted with WT H2A, H2AK119u1 and H2AK119u1I44A. Molecular weight markers are as indicated on the left. (**C**) Western blot of JARID2-1-530 purified after expression in insect cells. Molecular weight markers are as indicated on the left.

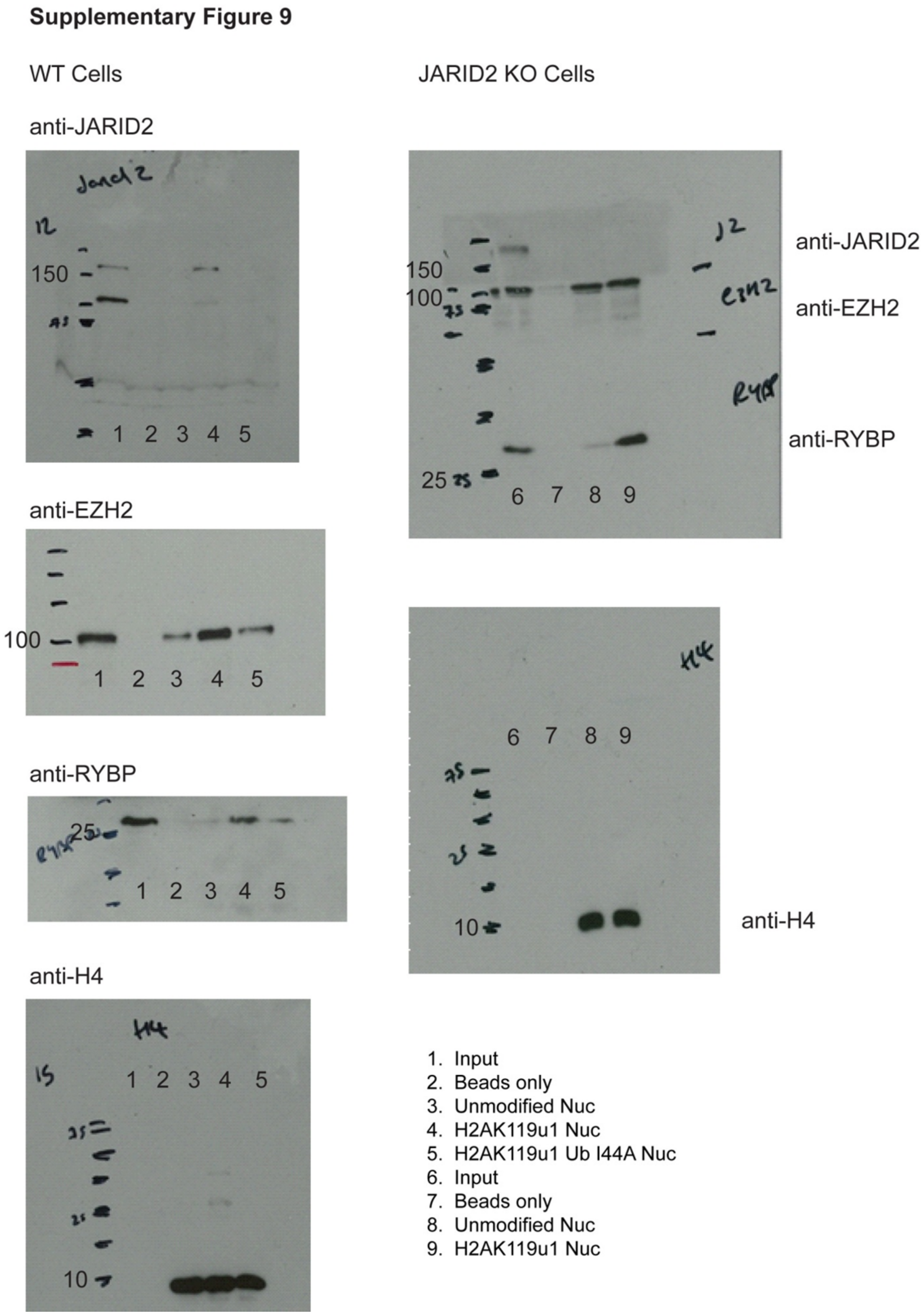

Supplementary Figure 9: Full Immunoblots for the experiment shown in Figure 4a.

Supplementary Table 1. Oligonucleotides used in this study

**Cloning primer**	**Sequence 5'- 3'**
J2F1	GTCGACATGAGCAAGGAAAGACCCAAGAGG
J2F121	GTCGACATGCAGCCGAATAGTCCCAGCAC
J2F542	GTCGACATGAAGGGGAGCGGCAAGTCTGGG
J2R1234	GCGGCCGCTCAGGCATAGTCAGGCACGTCATAAGGATAAACCTTTCTCTTCTTTTTTGGTGAGGATGGGAGCCGAGATGG
J2R583	GCGGCCGCTCAGGCATAGTCAGGCACGTCATAAGGATAAACCTTTCTCTTCTTTTTTGGCCCGTACTTCTCCACCTG
J2R541	GCGGCCGCTCAGGCATAGTCAGGCACGTCATAAGGATAAACCTTTCTCTTCTTTTTTGGCTCTGGCTTGCCCTGTGGG
**sgRNA target sites**	**Sequence (PAM in bold)**
Jarid2 (exon 3)	ATTTTGAAGAAGGGCCGTCG**AGG**
Atrx (exon 9)	ATGTCTTCTGGAACCGAGGA**AGG**
Suz12 (exon 7)	CCAATAAGACAAGTCCCTAC**TGG**
**KO validation primer**	**Sequence**
JARID2_HRMA_F2	CCCTTTTCTCACCTGTAGGG
JARID2_HRMA_R2	GATTCACACTTGCTCCCATGT
ATRX_HRM_F3	AGCTCTCCAGTTCTGGCTCAGTC
ATRX_HRM_R3	TAGTGGGGAACATAAGGGTTCAGG
SUZ12_HRM_F2	TCAATCTTTTCTTTGCCAGGAT
SUZ12_HRM_R2	CCAATATTTTCATTGGTTTCTCC
EZH2_HRM_R2	GCAAAAGATGGAGAATGTCTAAGGA
**Genotyping primer**	**Sequence**
JD2-5ARM-F	CCTTTATCTAGAACAGTAGTTCTCAGCC
JD2-3ARM-R	CACTCACTGGGTAGATTGATTACATAC
JD2-CASS-R	AGGAATGCCCAGCCAAAATC
FLP-F	CCCATTCCATGCGGGGTATCG
FLP-R	GCATCTGGGAGATCACTGAG
CRE-F	GCCTGCATTACCGGTCGATGCAACGA
CRE-R	GTGGCAGATGGCGCGGCAACACCATT
